# Synergistic effects of transcutaneous spinal stimulation and neuromuscular electrical stimulation on lower limb force production: Time to deliver

**DOI:** 10.1371/journal.pone.0296613

**Published:** 2024-08-30

**Authors:** Alexander G. Steele, Albert H. Vette, Catherine Martin, Kei Masani, Dimitry G. Sayenko

**Affiliations:** 1 Department of Neurosurgery, Center for Neuroregeneration, Houston Methodist Research Institute, Houston, Texas, United States of America; 2 Department of Mechanical Engineering, Donadeo Innovation Centre for Engineering, University of Alberta, Edmonton, Alberta, Canada; 3 Glenrose Rehabilitation Hospital, Alberta Health Services, Edmonton, Alberta, Canada; 4 Institute of Biomedical Engineering, University of Toronto, Toronto, ON, Canada; 5 KITE Research Institute–University Health Network, Toronto, ON, Canada; Fondazione Santa Lucia Istituto di Ricovero e Cura a Carattere Scientifico, ITALY

## Abstract

**Background:**

Transcutaneous spinal stimulation (TSS) and neuromuscular electrical stimulation (NMES) can facilitate self-assisted standing in individuals with paralysis. However, individual variability in responses to each modality may limit their effectiveness in generating the necessary leg extension force for full body weight standing. To address this challenge, we proposed combining TSS and NMES to enhance leg extensor muscle activation, with optimizing timing adjustment to maximize the interaction between the two modalities.

**Methods:**

To assess the effects of TSS and NMES on knee extension and plantarflexion force, ten neurologically intact participants underwent three conditions: (1) TSS control, (2) NMES control, and (3) TSS + NMES. TSS was delivered between the T10 and L2 vertebrae, while NMES was delivered to the skin over the right knee extensors and plantarflexors. TSS and NMES were administered using a 15 Hz train of three 0.5 ms biphasic pulses. During the TSS + NMES condition, the timing between modalities was adjusted in increments of ¼ the interval within a 15 Hz frequency, i.e., 66, 49.5, 33, 16.5, and 1 ms.

**Results:**

NMES combined with TSS, produced synergistic effects even on non-targeted muscle groups, thereby promoting leg extension across multiple joints in the kinematic chain. The sequence of NMES or TSS trains relative to each other did not significantly impact motor output. Notably, a delay of 16.5 to 49.5 ms between interleaved TSS and NMES pulses, each delivered at 15 Hz, results in more robust and synergistic responses in knee extensors and plantarflexors.

**Conclusions:**

By adjusting the timing between TSS and NMES, we can optimize the combined use of these modalities for functional restoration. Our findings highlight the potential of integrated TSS and NMES protocols to enhance motor function, suggesting promising avenues for therapeutic applications, particularly in the rehabilitation of individuals with SCI.

## Introduction

Spinal cord injury (SCI) is a life-long, devastating, and costly condition. Many individuals with SCI cannot stand by themselves due to paralysis of their leg muscles [1]. Affected individuals often spend a considerable amount of time in a seated position rather than engaging in standing activities, which has negative effects on mobility, autonomic functions, and quality of life [2]. Standing has various therapeutic benefits for individuals with SCI, such as improved blood circulation, respiration, bone density, and increased overall wellbeing [3]. For these reasons, upright standing is used as a primary therapeutic tool for individuals with SCI [4]. There are several assistive devices that enable individuals with SCI to stand upright, such as standing frames [5]. However, these tools only provide passive support and do not require additional effort from the individual. Yet, the benefits of standing therapy are maximized during active, self-assisted standing [4]. This is currently accomplished with some external support, such as walkers, knee-ankle-foot orthoses, or, more recently, powered exoskeletons, where weight-bearing is achieved primarily by the legs [6]. Active standing not only enhances muscle conditioning but it also promotes descending control of posture, such as during body-weight shifts. Considering the extensive reorganization of cortico-brainstem-spinal, corticospinal, and spinal networks following SCI [7–13], the functional improvement demonstrated during stand training will likely reflect the synergistic interactions within and between the supraspinal and spinal cord networks, contributing to improved rehabilitation outcomes.

Neuromodulation approaches promoting physiological responses in the lower limb and trunk muscles can be used in combination with activity-based therapies to potentiate functional outcomes. In this context, non-invasive Neuromuscular Electrical Stimulation (NMES), which uses electrical pulses to excite the peripheral motor nerves and generate contractions of paralyzed muscles, can augment or induce specific movements [14–16]. However, the use of NMES alone to promote standing requires it to be delivered and controlled over multiple muscle groups [17]. Transcutaneous Spinal Stimulation (TSS), another non-invasive electrical stimulation technique, has recently received much attention from clinicians and researchers [18–21]. TSS can produce motor responses via transsynaptic activation of motoneurons in multiple spinal segments and is capable of activating other neural components within the spinal cord, including interneurons, ascending sensory fibers in the dorsal columns, descending motor tracts, and other polysynaptic pathways [13, 22–26]. Thus, TSS can activate multiple paralyzed muscles at once and induce self-assisted standing in individuals with SCI while being delivered over the lumbar spinal cord [22]. However, the individual responses to TSS vary greatly depending, for instance, on the strength of the lower limb muscles, neural excitability, or asymmetry due to SCI; therefore, TSS may not sufficiently drive bilateral muscle activation for self-assisted standing for some individuals [22, 27]. We proposed that the integration of two neuromodulation methods, TSS and NMES, could unlock greater potential, offering amplified benefits from both neurophysiological and functional perspectives. Recognizing the shared neural pathways and targets of TSS and NMES, our objective was to enhance motor output in the leg extensors critical for standing by concurrently applying both modalities. In our investigation, we focused on mechanistically exploring the interaction between TSS and NMES in neurologically intact participants. Specifically, we applied trains of NMES and TSS, each at a frequency of 15 Hz, known to induce fused, tonic extensor activity specific for postural control in individuals with SCI [22, 28, 29]. We systematically explored the effects of various relative stimulation intervals (RSI) between NMES and TSS, each representing increments of one-quarter of the interval within a 15 Hz frequency, specifically 66, 49.5, 33, 16.5, and 1 ms. Based on the conduction time between the periphery and spinal cord, which ranges from approximately 10 to 20 ms [24, 25, 30], we suggested that pulses from NMES or TSS arriving at motoneurons projecting to the leg extensors, can modulate neuromuscular output depending on the timing of their convergence. Previous studies have demonstrated that NMES of the knee extensors or plantarflexors can alter spinal reflexes in lower limb muscles resulting in inhibition of the evoked responses [31, 32]. Conversely, other research has shown that post-activation potentiation can occur when descending volleys and electrically induced stimuli converge at the motor pools [23, 33–36]. Furthermore, the extent of activation of homonymous motor pools using different modalities of neurostimulation, and their interaction with heteronymous motor pools, remains poorly understood. This suggests that location, source, and timing of the stimuli can play key factors in the combined effect. The temporal interplay between NMES and TSS may depend on the antidromic or orthodromic transmission routes of NMES to the motor pool, as well as factors like the refractory period, post-tetanic potentiation, and the heteronymous effects of TSS on multisegmental motoneurons [26, 37, 38]. These factors can result in synergistic or antagonistic interactions. Therefore, our systematic investigation of the temporal dynamics between NMES and TSS at varying intervals considered the conduction time within the sensory and motor neural pathways from the spinal cord to lower limb muscles [23, 36, 39–41]. By measuring motor output generated by the lower limb extensors and spinally evoked motor potentials (SEMP) in the same muscles, our goal was to identify the optimal time range for synergistic force production.

In this light, we proposed that: (1) the combination of TSS and NMES can achieve greater activation of leg muscles than either modality alone; (2) the location of NMES will impact responses across the lower limb muscles; and (3) by adjusting the timing of stimuli, we can maximize the stimulation effect or observe inhibition. To quantitatively test these hypotheses, we separately examined the effects of combined TSS and NMES on knee extension and plantarflexion force generation in neurologically intact individuals, using different relative timing schemes. Additionally, SEMP were recorded from knee extensors and plantarflexors to quantify neuromuscular output so that it could be related to the observed force measurements.

## Methods

### Participants

Ten adults with no known neuromuscular or musculoskeletal impairments (6 females 4 males; age 27.5 ± 6 years, height 167.6 ± 7.6 cm, body mass 64.8 ±13 kg) agreed to participate in this study, and written informed consent was obtained from each of the participants. The experimental procedures were approved by the Houston Methodist Research Institute institutional review board (Study ID: Pro00020081) in accordance with guidelines established in the Declaration of Helsinki. The study did not include minors or people from vulnerable populations.

### Experimental setup

**Fig 1:**
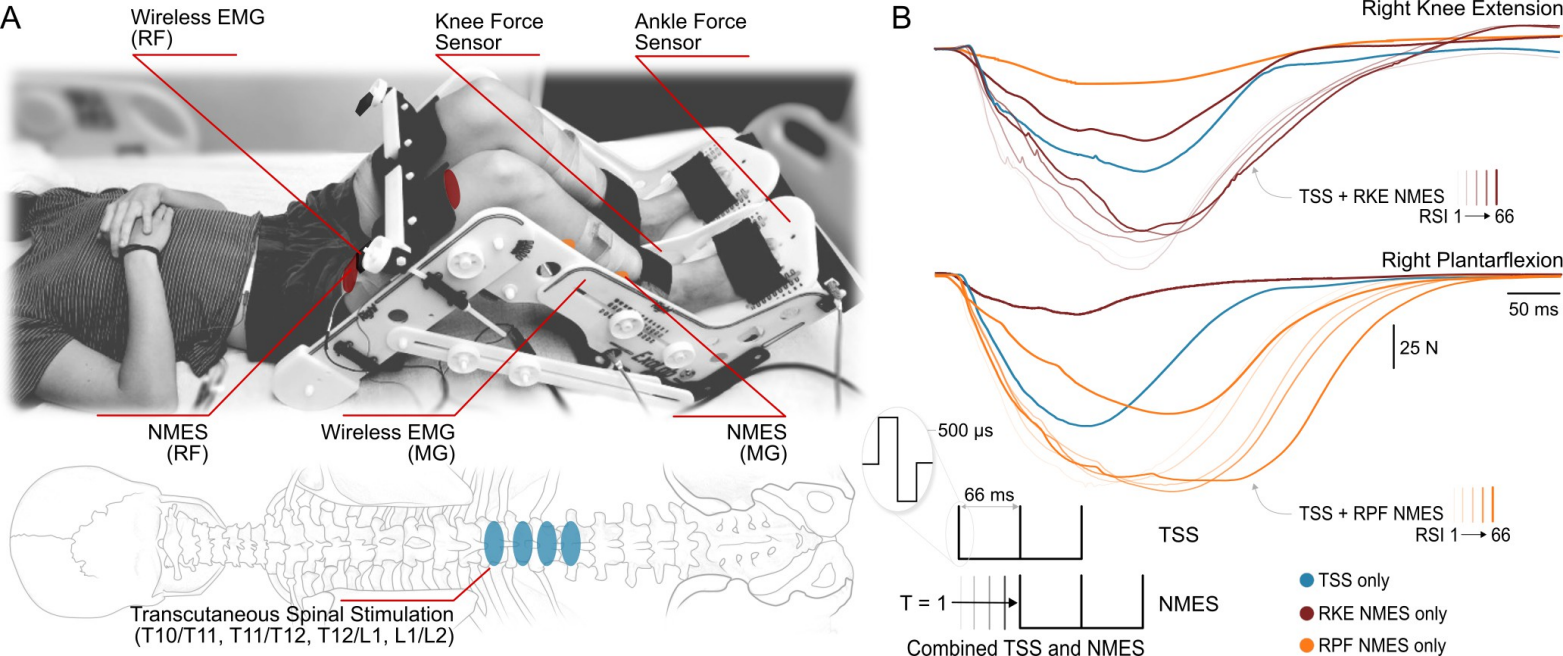
Experimental setup: (A) Transcutaneous spinal stimulation (TSS) was applied between the T10/T11 through L1/L2 spinous processes. Neuromuscular electrical stimulation (NMES) was delivered to the right knee extensors or plantarflexors. EMG was recorded bilaterally from the rectus femoris (RF) and medial gastrocnemius (MG) muscles. (B) Electronically controlled stimulation was delivered using biphasic triplet pulses with a 66 ms inter-pulse interval (i.e., at 15 Hz). TSS and NMES were delivered at different relative stimulus intervals (RSI) ranging T = 1 to 66 ms, indicating TSS preceded NMES and from -1 to -66 ms, indicating NMES preceded TSS. Where RSI is the time delay of the first pulse in the NMES triplet relative to the first pulse in the TSS triplet. Blue traces denote forces during TSS control, thick brown traces denote forces during NMES only of the right knee extensors (RKE), and thick orange traces denote forces during NMES only of the right plantarflexors (RPF). Note that while force sensors were independent for the knee and ankle joints, NMES delivered to either knee extensors or plantarflexors inevitably caused changes in the measured forces from a synergistic joint. Shaded traces denote force response as RSI increase from RSI 1 (lightest) to RSI 66 (darkest) for TSS + NMES of the right knee extensors (thin brown) and right plantarflexors (thin orange).

TSS was delivered to the skin over the lumbosacral spinal enlargement using an electronically triggered constant current stimulator DS8R (Digitimer Ltd., UK) via self-adhesive electrodes (PALS, Axelgaard Manufacturing Co. Ltd., USA). Four cathodes (diameter: 3.2 cm) were placed along the midline of the spine between the spinous processes starting between the T10 and T11 vertebrae and ending between the L1 and L2 vertebrae as seen in Fig 1A. Spinal levels were determined via palpation. Two oval anodes (size: 7.5 cm x 13 cm) were placed bilaterally and symmetrically on the abdomen [42–44]. NMES was delivered to the skin over the right knee extensors and plantarflexors only, using a second constant current stimulator DS8R (Digitimer Ltd., UK). The cathodes and anodes (size: 7.5 cm x 13 cm) were placed on the proximal and distal aspects of each muscle group, respectively. Both TSS and NMES were delivered using a train of three 0.5 ms biphasic square-wave pulses at 15 Hz (e.g., with an inter-stimulus interval of 66 ms within each train). Triplets were chosen to induce bursts, similar to functional stimulation delivery when fused muscle contractions promote movements [45].

Trigno Avanti wireless surface electromyography (EMG) electrodes (Delsys Inc., USA) were placed longitudinally over the right rectus femoris (RF) and medial gastrocnemius (MG) muscles. EMG data were amplified using a Trigno Avanti amplifier (Delsys Inc., USA).

The tests were performed in supine position with the participants’ legs placed in the Exolab apparatus (Antex Lab LLC, Russia) supporting the hip, knee, and ankle joints at 155, 90, and 90 degrees, respectively (Fig 1A) [42, 43]. These joint angle positions were selected to isolate knee and ankle joint movements. Four calibrated, two-sided load cells (FSH00007, Futek, USA) measured forces generated by the knee extensors and plantarflexors independently. EMG and force signals were recorded at a sampling frequency of 2,000 Hz using a PowerLab data acquisition system (ADInstruments, Australia).

### Experimental procedure

To determine the motor threshold and maximal response, recruitment curves were generated by increasing the TSS amplitude in 5 mA increments, beginning with 30 mA. The NMES was delivered with an amplitude beginning at 5 mA and was increased in 1 mA increments. The maximal response was defined as two successive increases in stimulation amplitude where the median peak-to-peak response did not change by more than two standard deviations, and the motor threshold was defined as the first evoked response that was greater than two standard deviations from baseline. Three triplets of each amplitude were delivered in succession via an electronically triggered stimulator, with a minimum of 2 seconds between triplets, until the force generated by the knee extensors and plantarflexors plateaued or reached the maximum tolerated amplitude. Then, the TSS amplitude was reduced to generate approximately 50% of the maximum force produced by the knee extensors or plantarflexors using the data collected from the recruitment curve procedure. Subsequently, the NMES amplitude for the knee extensors or plantarflexors was adjusted independently to match the force generated by the target muscle group at the selected TSS amplitude. Fig 1B outlines the experimental paradigm. The TSS + NMES condition was performed in two separate blocks: first, where TSS was paired with NMES of the knee extensors, and second, where TSS was paired with NMES of the plantarflexors. Before and after the TSS + NMES blocks, control responses to TSS only and NMES only were recorded to account for possible changes of motor output over the course of the experiment. The RSI during TSS + NMES was defined as the time between the first TSS pulse and the first NMES pulse in the triplets. Because the RSI are relative to the first TSS pulse, a negative RSI indicates that NMES preceded TSS, while positive RSI indicate that NMES was delivered after TSS. The RSI was varied using 16.5 ms increments via electronic control of the timing between the two stimulators. The order of RSI was randomized, and each RSI was repeated three times in succession. The time between repetitions was randomized with a minimum of two seconds between repetitions of the same RSI and a minimum of five seconds between different RSI. Negative RSI were used so that the conditioned SEMP could be visualized in all recorded muscles, including the muscle groups receiving NMES. These data were used to quantitatively characterize the potentiation or depression of the SEMP, rather than inferring these changes from force production [33, 42–44]. To minimize the influence of volitional effort, we continuously monitored the EMG signals from the leg muscles in real-time throughout the experiment.

### Analysis

To evaluate the synergistic effects between produced joint forces, the right knee extension and plantarflexion force data were analyzed regardless of which muscles NMES was applied to. First, the integral of the force produced by the knee extensors and plantarflexors was averaged for each RSI at both the knee and ankle joints independently. Because force output depends on several variables (e.g., height, weight, stimulation amplitudes, etc.), the data were normalized to the TSS control within each participant. This normalization ensured that the average force produced during each participant’s TSS controls was set to zero, allowing for group comparisons. Consequently, a negative difference represents a decrease in produced force for a given RSI compared to the forces produced during the TSS condition. Additionally, the peak-to-peak amplitude of SEMP in the right RF and MG in response to the first TSS pulse following NMES was calculated at RSIs -66, -49.5, and -33. Due to the proximity of the EMG recording electrodes to the NMES electrodes, substantial long-lasting NMES artifacts can saturate the recordings, masking the SEMP. Therefore, the evoked motor responses at RSI other than those listed above were not included in the analysis (see S1 Dataset and S1 File for examples).

All statistical analyses were performed using the open-source software, R (version 4.3.3, Vienna, Austria) and RStudio (version 1.4.1717, RStudio PBC, Boston, MA). Normality was tested with a Shapiro-Wilk test and because normality was violated, a Kruskal–Wallis test was performed. Post hoc comparisons were performed using a Dunn test and a Bonferroni correction was applied to account for multiple comparisons. First, we compared changes in force production during various RSI in relation to the NMES control. Next, changes in force production were compared within positive and negative RSI. Finally, the average force production was compared between the negative and positive RSI. Using the EMG data, changes in the SEMP amplitude were compared at different RSI and to the TSS control. All p values reported are the p_adj_ values obtained after the Bonferroni correction was applied. For all performed comparisons a p_adj_ < 0.05 was considered significant (* p_adj_ < 0.05 and ** p_adj_ < 0.01).

## Results

### Produced forces

The recruitment curve procedure with TSS yielded maximum force generation of 22.0 ± 13.0 N by the knee extensors and 50.3 ± 26.8 N by the plantarflexors. The median force generated by the knee extensors and plantarflexors during the TSS control was 13.4 ± 6.8 N and 36.1 ± 10.1 N, respectively. There was no difference between the generated forces during the pre- and post-condition controls for either joint. During the TSS and NMES control trials, the average TSS intensity was 71.5 ± 14.5 mA (median ± S.D.), while the average intensity of NMES applied to the knee extensors and plantarflexors was 21.9 ± 9.6 mA and 12.9 ± 5.4 mA, respectively.

**Fig 2:**
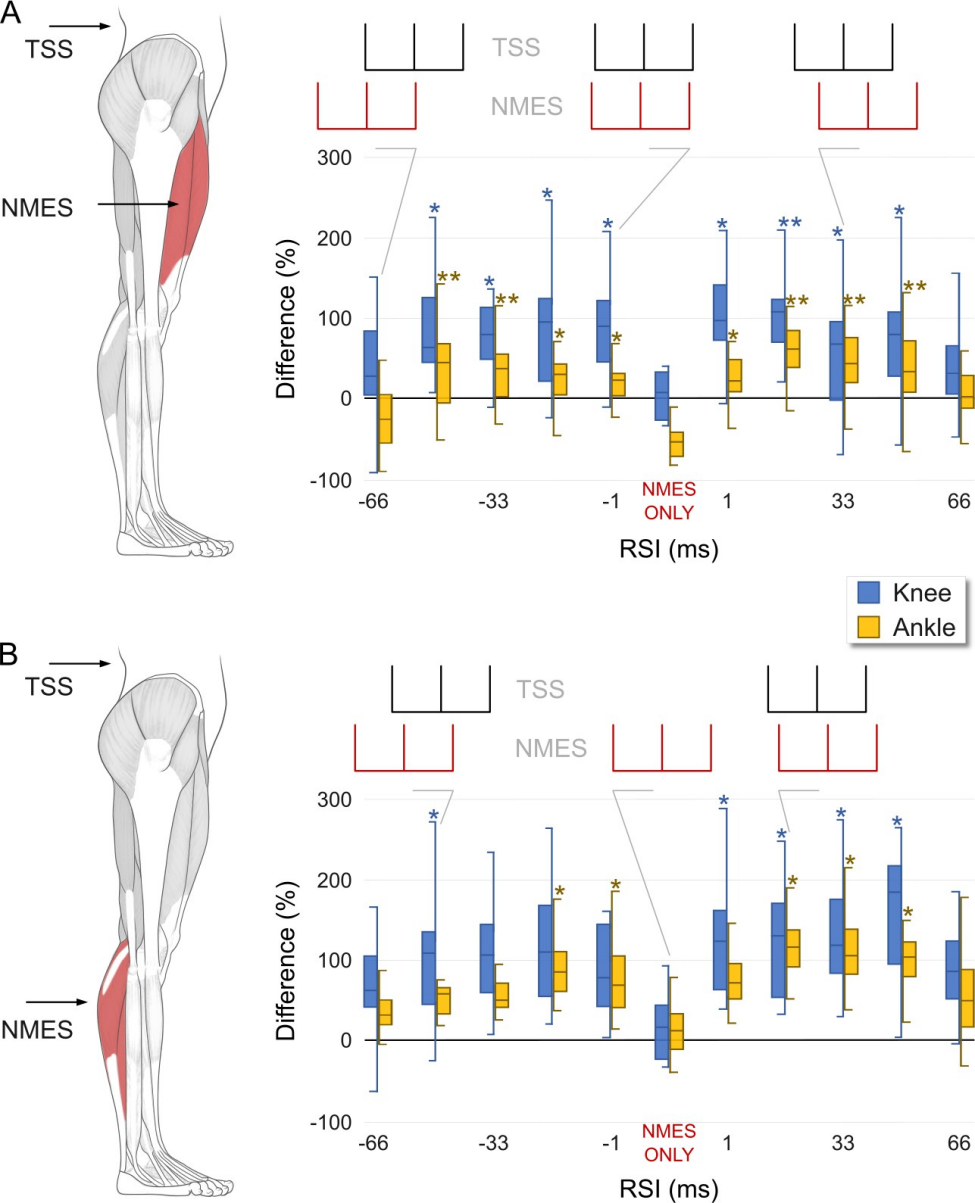
Produced forces: The differences in the force-time integral measured as area under the curve, for the right knee extensors (blue) and right plantarflexors (yellow) during TSS + NMES and NMES only, as the relative stimulation interval (RSI) changes during the (A) NMES applied to the knee extensors and (B) NMES applied to the plantarflexors conditions. Median and interquartile differences in the force-time integral were normalized to the TSS only condition, where 0% corresponds to the average force produced during the TSS control. Negative RSI indicate that NMES was delivered prior to TSS, while positive RSI indicate that TSS was delivered prior to NMES. Asterisks denote significant (*p < 0.05, **p < 0.01) changes compared to the NMES control. Triplet waveforms depicting different RSI are shown above each plot with TSS in black and NMES in red.

Fig 2 illustrates the dynamic changes in the knee extension and plantarflexion force-time integral during the combined TSS + NMES protocol. NMES was applied to the knee extensors (Fig 2A) and plantarflexors (Fig 2B) at various RSIs relative to TSS. Notably, changes in motor output were observed in both the knee extensors and plantarflexors, regardless of which muscle group was stimulated or the sequence of TSS and NMES applications. During TSS and NMES of the knee extensors, the produced knee extension force showed main effects for both negative (X^2^(5) = 15.557, p < 0.01) and positive (X^2^(5) = 17.715, p < 0.01) RSIs. Similarly, the produced plantarflexion force also showed main effects for both negative (X^2^(5) = 16.748, p < 0.01) and positive (X^2^(5) = 16.735, p < 0.01) RSIs (Fig 2A). There were no differences in average force production at either joint between the negative and positive RSIs. It is worth noting that relatively higher knee extension force was generated during RSIs of -16.5 and 16.5, although this increase was not significantly different from other RSIs.

During TSS and NMES of the plantarflexors, the produced plantarflexion force yielded main effects for both negative (X^2^(5) = 21.03, p < 0.001) and positive (X^2^(5) = 13.083, p < 0.05) RSIs. Additionally, the produced knee force also showed main effects for both negative (X^2^(5) = 14.228, p < 0.01) and positive (X^2^(5) = 14.228, p < 0.01) RSIs. The average force production by the plantarflexors was higher during positive RSIs compared to negative RSIs (p < 0.05). Relatively higher plantarflexion force was generated during RSIs of -16.5 and 16.5, although this increase was not significantly different from other RSIs, except of RSI -66. It is worth noting that RSI -66 yielded the lowest gain of plantarflexion force within the negative RSIs (RSIs -1, -16: p < 0.01; RSI -33, -49.5: p < 0.05).

### Spinally evoked motor potentials at RSIs -66, -49.5, and -33

**Fig 3:**
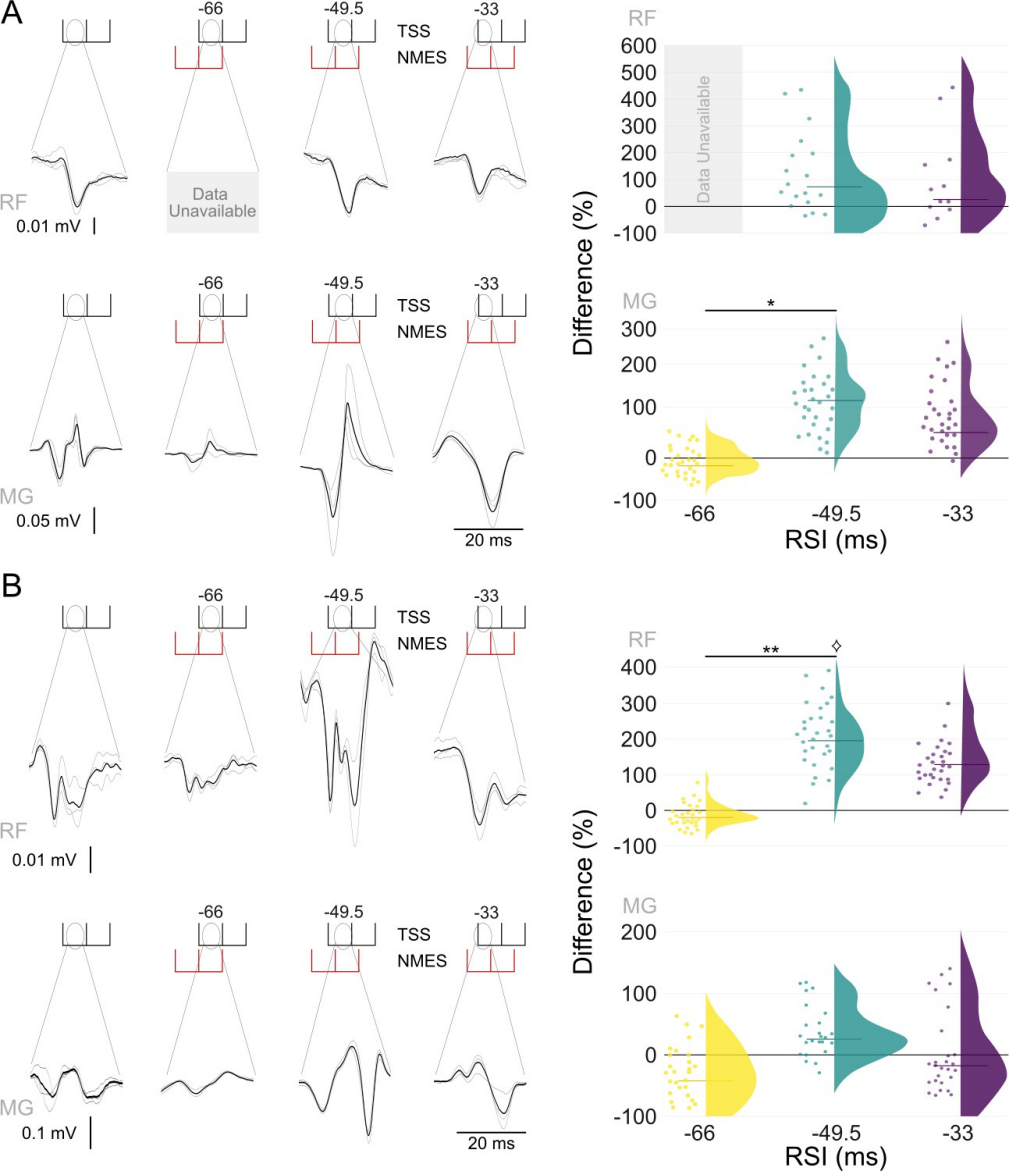
Changes in peak-to-peak amplitude of SEMP in the RF and MG muscles during the TSS control and TSS preceded by NMES, when (A) NMES is applied to the knee extensors and (B) to the plantarflexors for a representative participant (left panels). Individual responses are shown in light grey, with the average in black. The response distribution in the group data (right panels) is shown for RSIs -66, -49.5, and -33. Each dot represents an individual participant data point, and a half violin with a line denotes the median. Data were normalized to average peak-to-peak response during the TSS control within each participant, and are presented as the percent difference between TSS control and NMES+TSS. Asterisks denote significant (* p < 0.05 and ** p < 0.01) differences between RSIs. ⟡ denotes significant differences relative to the TSS control.

Fig 3 illustrates individual SEMPs in the RF and MG (left panel) along with the group data (right panel) during TSS preceded by NMES applied to the knee extensors and plantarflexors at different RSIs. Individual waveforms exemplify both inhibition (e.g., MG during NMES of the knee extensors at RSI -60) and facilitation (e.g., RF during NMES of the plantarflexors at RSI -49.5) of SEMPs. When NMES was applied to the knee extensors prior to TSS, group data analysis revealed main effects for the MG SEMP amplitude across different RSIs (X^2^(2) = 8.849, p < 0.05), with no significant effects observed in the RF (Fig 3A). Post-hoc comparisons revealed a difference in the MG SEMP amplitude between RSI -66 and RSI -49.5. However, changes in both the RF and MG SEMP amplitudes were not significant at any RSIs when compared to the TSS control.

When NMES was applied to the plantarflexors prior to TSS, main effects were found for the RF SEMP amplitude across different RSIs (X^2^(2) = 9.0606, p < 0.01) (Fig 3B). Post-hoc comparisons identified a significant difference in the RF SEMP amplitude between RSI -66 and RSI -49.5 (p < 0.01), with facilitation of the RF SEMP at RSI -49.5 compared to the TSS control (p < 0.05). No significant differences in the MG SEMP amplitude were observed across various RSIs or when compared to the TSS control.

## Discussion

To elucidate the interaction between TSS and NMES on leg muscle force generation, we integrated TSS targeting the lumbar spine with NMES applied to either the knee extensors or plantarflexors in neurologically intact individuals at rest. Our findings indicate that the combined TSS and NMES protocols led to increased force production in both the knee extensors and plantarflexors, independent of the NMES site and across various RSIs.

The sequential application of TSS and peripheral nerve stimulation has been investigated in prior studies involving both neurologically intact participants and individuals with SCI [46–49]. These studies aimed to understand the interactions between afferent signals, spinal neuronal networks, and resulting efferent muscle activation. The results revealed distinct and reproducible modulations in SEMP induced by TSS, with suppression of amplitude observed, particularly in ipsilateral proximal and distal flexor and extensor muscle groups [50]. The attenuation of motoneuron pool excitability following conditioning peripheral nerve stimulation was evident even when the latter was delivered at sub-motor threshold amplitude.

In our study, which involved overlapping patterns of TSS and NMES, we primarily observed synergistic and facilitatory effects on force generation by the lower limb extensors (Fig 2). These effects were notably enhanced when TSS and NMES, each delivered at a frequency of 15 Hz, were interleaved to create a combined pulse frequency higher than that of each modality applied individually. This likely contributed to the overall increase in force observed. For instance, delays of 16.5, 33, or 49.5 ms between the NMES and TSS 15 Hz pulses, appeared more effective in enhancing force output compared to longer delays such as RSI -66 (or 66), where the net frequency of TSS + NMES remained closer to 15 Hz.

Visual observation and group analysis of changes in the SEMP amplitude in the knee extensors and plantarflexors during TSS preceded by NMES indicated both faciliatory and inhibitory effects when these two stimulation modalities were combined. These effects were dependent on the delay between the NMES and TSS pulses, revealing decreased or unchanged SEMP amplitude during RSI -66 and increased responses during RSIs -49.5 and -33. Although this timing dependence of NMES and TSS stimuli interaction is not consistent with the conduction time between the periphery and spinal cord, projections within multisegmental motor pools, such as RF and MG, may contribute to these effects. The presence of stimulation artifacts from NMES prevented a systematic exploration of its conditioning effects on the SEMPs induced by TSS (see S1 Dataset and S1 File). Consequently, the effects of other RSIs, such as 16.5 ms or 1 ms, from NMES to SEMP remain to be investigated in future studies.

Previous research has shown that the bipedal stance of humans may lead to a contextual selection of heteronymous Ia patterns, possibly mediated by presynaptic inhibition directed at specific motoneurons [51, 52]. Our data suggest that volleys from NMES applied to the extensors involved in standing can modulate the excitability of their synergists, although the muscles directly stimulated by NMES were not affected. For instance, when NMES was applied to the knee extensors, modulation of SEMP could be seen in MG, and when NMES was applied to the plantarflexors, changes were observed in RF. These observations concur with previous studies proposing mechanisms of heteronymous Ia excitation produced by NMES [31, 51–54]. Further research should characterize this effect to determine optimal strategies for promoting this synergy, which may be beneficial for restoring self-supported standing and walking in individuals with SCI.

From a functional perspective, our data convey several messages: 1) Due to the synergistic action during leg extension or standing, NMES of either the knee extensors or plantarflexors, when delivered in combination with TSS, will have synergistic effects on the non-targeted muscle group, promoting leg extension in multiple joints of the kinematic chain; 2) The sequence of NMES or TSS trains in relation to each other does not significantly impact motor output, which is practical for continuous stimulation settings; 3) A delay within the range of 16.5 to 49.5 ms between interleaved NMES and TSS pulses, each delivered at 15 Hz, can yield more robust and synergistic responses in knee extensors and plantarflexors.

### Limitations

Despite the insightful findings presented in this study, certain limitations warrant consideration. Firstly, the inclusion of neurologically intact participants, as opposed to individuals with SCI, may limit the generalizability of the results to the target population. Additionally, the experimental setup involved participants in a supine position rather than standing or engaging in other functional activities. While this choice was made to control variables, it does introduce a potential limitation regarding the translation of the observed effects to weight-bearing scenarios. Further validation of the observed synergistic interaction of TSS and NMES in real-world standing scenarios among individuals with SCI is essential for a more comprehensive understanding of the clinical implications of our findings. Furthermore, the investigation into the mechanisms underlying the combination of NMES and TSS was hindered by confounding stimulating artifacts in EMG data. These artifacts posed challenges in obtaining a detailed understanding of the interactions between NMES and TSS, highlighting the need for further research to delineate the specific mechanisms driving their combined effects. TSS was delivered with four electrodes in the interspinous spaces between T10 and L2. While we believe the synergistic effects of TSS and NMES are illustrative regardless of the number of electrodes used for TSS, future investigations may explore potential effects of using fewer electrodes within the specified area. The triplet-based stimulation approach, with its relatively brief duration of force generation in the NMES+TSS condition, may present challenges in directly extrapolating the findings to sustained standing scenarios. The choice to use the triplet pulse was made primarily so that the underlying mechanisms of combined TSS and NMES could be explored at intensities higher than the motor threshold, a crucial aspect in individuals with intact sensation during TSS at 15 Hz or more, commonly used for facilitating standing in individuals with SCI. While this study was limited to 15 Hz stimulation, future work using other frequencies may provide additional insights into the interaction between TSS and NMES.

## Conclusion

We investigated the interaction between TSS and NMES on force generation and motor responses in the leg muscles. Our findings highlight several key implications: NMES of either the knee extensors or plantarflexors, when combined with TSS, produces synergistic effects on non-targeted muscle groups, promoting leg extension across multiple joints. Implementing a delay of 16.5 to 49.5 ms between interleaved NMES and TSS pulses, each delivered at 15 Hz, results in more robust and synergistic responses in knee extensors and plantarflexors. These insights underscore the potential of combined TSS and NMES protocols to improve motor function, suggesting promising avenues for therapeutic applications, particularly in the rehabilitation of individuals with SCI. Further research should aim to optimize these interventions and explore their long-term benefits in clinical settings.

## Supporting information

S1 Dataset This ZIP file contains the data used for the statistical analysis. Each file name is by the order of stimulation. For example, if right knee extensors were stimulated using NMES prior to the application of TSS (i.e., the negative RSI), the file name will start with RKEtoTSS. Data are further divided by force and EMG data. Files containing force data will contain -Force at the end of the file name while EMG data will contain either -RRF or -RMG signifying SEMP data from right RF or right MG, respectively. RSI are contained in the “Delay” column. Force data will contain a Delay value of 0, which signifies the NMES-only condition.
(ZIP)

S1 File This document contains examples of TSS and NMES artifacts and demonstrates why some evoked responses were not recoverable. (DOCX)
