# Supplementary material for: Synergistic effects of transcutaneous spinal stimulation and neuromuscular electrical stimulation on lower limb force production: Time to deliver
Source: PLoS One. 2024 Aug 30;19(8):e0296613. doi: 10.1371/journal.pone.0296613 (PMC11364223; doi:10.1371/journal.pone.0296613)
Supplement: S1 File — (DOCX) [file pone.0296613.s002.docx]

**Supplementary**

***TSS+NMES artifacts***

The large NMES artifacts recorded from the rectus femoris (RF) obscure the evoked response across various RSIs. Figure 1A-C demonstrates the recorded responses in RF during TSS and NMES of the knee extensors, delivered at relative stimulation intervals (RSIs) of 1, 16.5, and 33 ms. Due to the NMES artifact, electrically induced muscle responses could not be seen. Figure 1D-E provides a detailed explanation of these effects. Specifically, during TSS only, the spinally evoked motor potential (SEMP) occurs at a latency of approximately 15.3 ms (Fig. 1D). However, when NMES precedes TSS at an RSI of 1 ms, no SEMP is visible due to the recovery time of the NMES stimulation artifact. While the first SEMP may be obtained following NMES at longer RSIs (e.g., an RSI of 16.5 ms and above), these SEMPs will be unconditioned. Therefore, negative RSIs were used during the experiment to provide information regarding the behavior of the conditioned SEMP during preceding NMES.


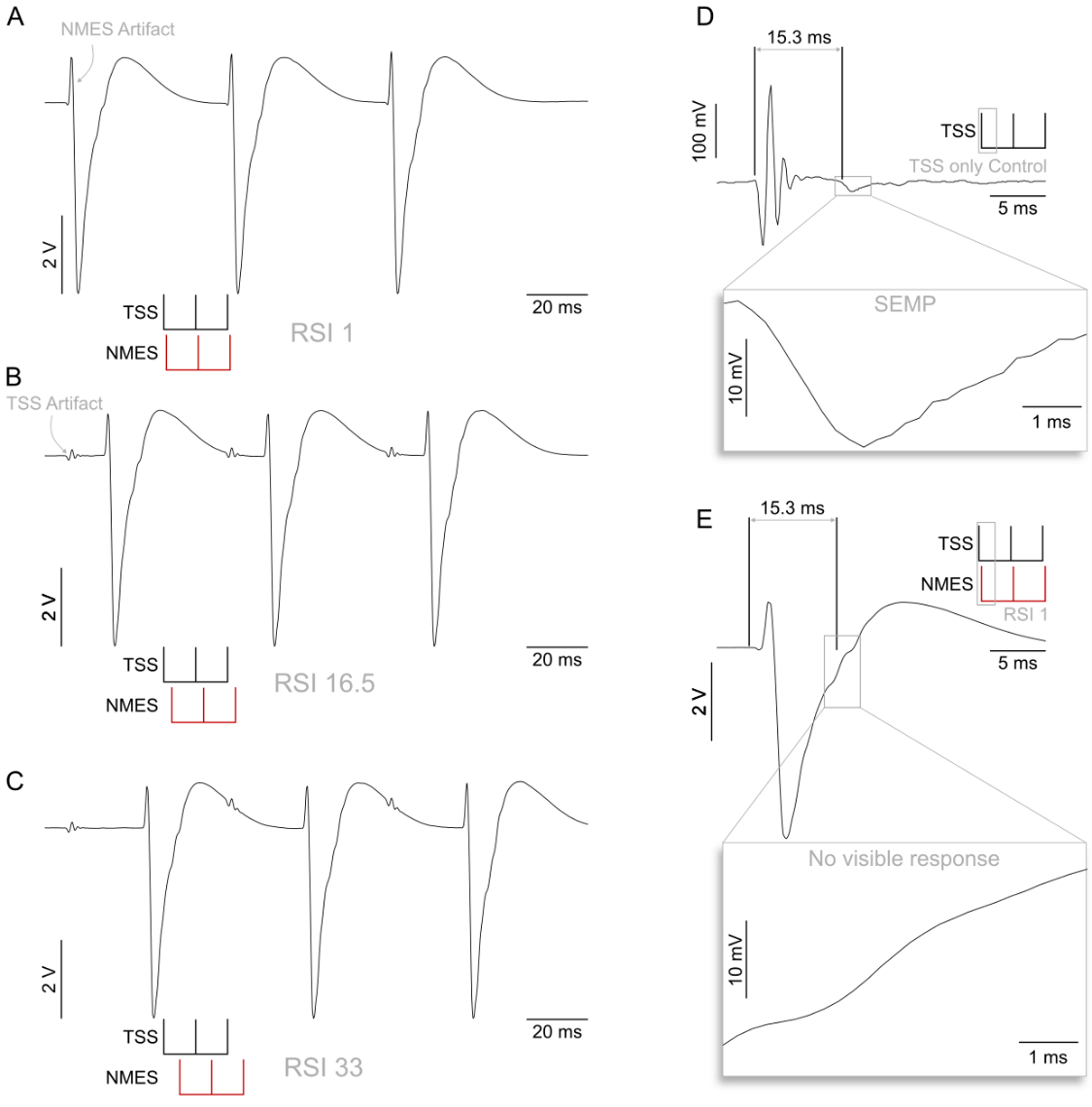


**Figure S1 TSS+NMES condition.** When NMES is applied to the knee extensors, the EMG recorded from the RF becomes “contaminated” with the stimulation artifact. This can be seen (A) at RSI 1, (B), RSI 16.5, and (C) RSI 33 ms. For comparison, the TSS only condition (D) induced a visible SEMP recorded from RF with a latency of approximately 15.3 ms. However, during the TSS+NMES at an RSI of 1 ms, no visible response was observed at the expected latency of 15.3 ms in relation to the TSS stimulation.
